# Eight Weeks of a High Dose of Curcumin Supplementation May Attenuate Performance Decrements Following Muscle-Damaging Exercise

**DOI:** 10.3390/nu11071692

**Published:** 2019-07-23

**Authors:** Ralf Jäger, Martin Purpura, Chad M. Kerksick

**Affiliations:** 1 Increnovo LLC, 2138 East Lafayette Place, Milwaukee, WI 53202, USA; 2 Exercise and Performance Nutrition Laboratory, Lindenwood University, St. Charles, MO 63301, USA

**Keywords:** athletic performance, muscle-damaging exercise, recovery, downhill run

## Abstract

Background: It is known that unaccustomed exercise—especially when it has an eccentric component—causes muscle damage and subsequent performance decrements. Attenuating muscle damage may improve performance and recovery, allowing for improved training quality and adaptations. Therefore, the current study sought to examine the effect of two doses of curcumin supplementation on performance decrements following downhill running. Methods: Sixty-three physically active men and women (21 ± 2 y; 70.0 ± 13.7 kg; 169.3 ± 15.2 cm; 25.6 ± 14.3 body mass index (BMI), 32 women, 31 men) were randomly assigned to ingest 250 mg of CurcuWIN® (50 mg of curcuminoids), 1000 mg of CurcuWIN® (200 mg of curcuminoids), or a corn starch placebo (PLA) for eight weeks in a double-blind, randomized, placebo-controlled parallel design. At the end of the supplementation period, subjects completed a downhill running protocol intended to induce muscle damage. Muscle function using isokinetic dynamometry and perceived soreness was assessed prior to and at 1 h, 24 h, 48 h, and 72 h post-downhill run. Results: Isokinetic peak extension torque did not change in the 200-mg dose, while significant reductions occurred in the PLA and 50-mg groups through the first 24 h of recovery. Isokinetic peak flexion torque and power both decreased in the 50-mg group, while no change was observed in the PLA or 200-mg groups. All the groups experienced no changes in isokinetic extension power and isometric average peak torque. Soreness was significantly increased in all the groups compared to the baseline. Non-significant improvements in total soreness were observed for the 200-mg group, but these changes failed to reach statistical significance. Conclusion: When compared to changes observed against PLA, a 200-mg dose of curcumin attenuated reductions in some but not all observed changes in performance and soreness after completion of a downhill running bout. Additionally, a 50-mg dose appears to offer no advantage to changes observed in the PLA and 200-mg groups.

## 1. Introduction

Even in trained athletes, a novel or unaccustomed exercise bout, especially those that emphasize eccentric contractions, can cause microscopic intramuscular tears and an exaggerated inflammatory response [1,2], which is generally referred to as exercise-induced muscle damage (EIMD) [3]. The subsequent muscular pain and restriction of movement from EIMD can limit an athlete’s performance [4]. Thus, strategies that can attenuate performance decrements associated with EIMD should result in higher training quality and hypothetically greater exercise training adaptations. Consequently, many strategies have been proposed to treat or prevent EIMD [5], but there is still no scientific consensus on the most effective strategy for all individuals [6].

Curcumin, the bioactive component (2–5% by weight) of the spice herb turmeric, has a long history of medicinal use due to its anti-inflammatory and antioxidant properties [7]. The United States Food and Drug Administration has listed curcumin as GRAS (generally recognized as safe), and curcumin-containing supplements have been approved for human ingestion [8]. Curcumin is a polyphenol that is considered to be a “nutraceutical”, or a dietary agent with pharmaceutical or therapeutic properties. The capacity for curcumin as a treatment strategy in favor of targeted pharmaceuticals is promising due to curcumin being a highly pleiotropic molecule that interacts with multiple inflammatory pathways [1,2].

Previous research has indicated that curcumin may help alleviate performance decrements following intense, challenging exercise. For example, initial research in mice indicated that curcumin supplementation led to greater voluntary activity and improved running performance compared to placebo-supplemented mice after eccentric exercise [9]. Similar effects of curcumin following EIMD have been reported in human subjects. One study reported that curcumin supplementation reduces pain and tenderness [10], while Drobnic et al. [11] reported a reduction in muscular trauma in the posterior and medial thigh following a downhill run with curcumin supplementation along with a moderate reduction in pain. In contrast, Nicol et al. [12] reported that curcumin moderately reduced pain during exercise, but had little effect on muscle function. Relative to the impact of specific curcuminoids (as opposed to curcumin supplementation) in response to exercise and muscle damage, no published literature is available at the current time. A systematic review by Gaffey et al. [13] concluded that insufficient evidence exists to support the ability of curcuminoids to relieve pain and improve function. Importantly, the authors highlighted a general lack of evidence and poor study quality from the existing literature base.

The mixed findings in previous studies may have been a result of the limited bioavailability due to formulation of the supplement [14]. A major limitation to the therapeutic potential of curcumin is its poor solubility, low absorption from the gut, rapid metabolism, and systemic elimination [15]. Curcumin is primarily excreted through the feces, never reaching detectable levels in circulation [16]. Previously, it has been shown that high doses of orally administered curcumin (e.g., 10–12 g), has resulted in little-to-no appearance of curcuminoids in circulation [17]. Various methods have been developed to increase the bioavailability of curcumin involving emulsions, nanocrystals, and liposomes, with varying degrees of success [18]. A recent formulation of curcumin (CurcuWIN®) involves the combination of a cellulosic derivatives and other natural antioxidants (tocopherol and ascorbyl palmitate). This formulation has been shown previously to increase absorption by 45.9-fold over a standardized curcumin mixture and a 5.8 to 34.9-fold greater increase in absorption (versus other formulations) while being well tolerated with no reported adverse events [19].

As part of participating in regular exercise training programs, much interest exists to maximize strategies to reduce decrements in performance and improve training quality. As a strategy to reduce soreness, loss of muscle function, and inflammation, curcumin serves as a potentially useful nutritional target to aid in accomplishing these goals, yet longstanding shortcomings with the ingredient have limited its potential. Therefore, the purpose of this study was to examine, in a double blind, placebo-controlled fashion, whether a novel form of curcumin would attenuate performance decrements and reduce inflammation following a downhill running bout. Beyond examining the impact of curcumin availability, an additional study question focused upon the identification of dose-dependent outcomes associated with curcumin administration relative to its ability to attenuate performance changes. We hypothesize that in a dose-dependent fashion, curcumin will attenuate changes in performance indicators after completion of a downhill bout of treadmill running.

## 2. Materials and Methods

The data reported in this study were collected as part of a randomized, double-blind, placebo-controlled investigation that examined the effects of eight weeks of curcumin supplementation on blood flow, exercise performance, and muscle damage. The data involving blood flow have already been published [20]. This study was conducted in accordance with the Declaration of Helsinki guidelines and registered with the ISRCTN registry (ISRCTN90184217). All the procedures involving human subjects were approved by the Institutional Review Board of Texas Christian University for use of human subjects in research (IRB approval: 1410-105-1410). Written consent was obtained from all the participants prior to any participation.

### 2.1. Participants

Men and women aged 19 to 29 years were considered eligible for participation if he or she was a non-smoker and free of any musculoskeletal, medical, or metabolic contraindications to exercise. The health status and activity levels of potential participants were determined by completion of a medical history form and a physical activity record. Further, participants had to be low to moderately trained, which was defined as meeting the current American College of Sports Medicine (ACSM) guidelines of at least 150 min of moderate aerobic activity, or 75 min per week of vigorous aerobic activity per week for at least the past three months [21]. Exclusion criteria included women who were pregnant or lactating, any participation in another clinical trial or consumption of an investigational product within the previous 30 days, receiving regular treatment with anti-inflammatory/analgesic/antioxidant drugs in the previous month, or the use of any ergogenic aid during the nine-week period prior to recruitment.

A total of 106 participants (53M, 53F) were initially screened for study participation. Of this cohort, 32 additional participants (15M, 17F) were excluded from study involvement due to them not meeting the study’s inclusion or exclusion criteria. Consequently, a total of 74 subjects were randomly assigned to a supplementation group and from there, an additional 11 participants (7M, 4F) withdrew from the study due to different priorities, i.e., school, resulting in a total of 63 (31M, 32F) study participants who completed the study. The subjects’ characteristics are presented in Table 1.

### 2.2. Experimental Protocol

**Figure 1:**
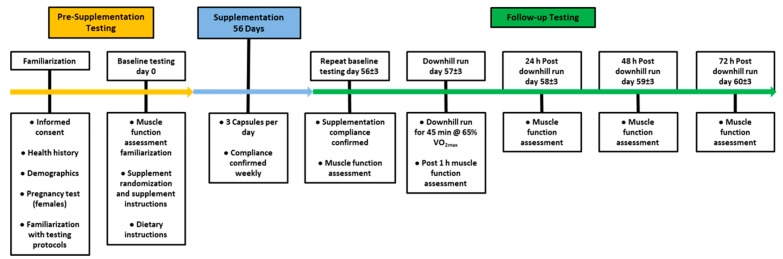
Study Design. Measurements are noted along the time line.

A summary of the study design is presented in Figure 1. All the testing was performed in the Exercise Physiology Laboratory within the Kinesiology Department at Texas Christian University. Prior to supplementation, and 3 to 7 days after a familiarization visit, participants returned to the laboratory after having abstained from any strenuous physical activity for at least 48 h. Additionally, participants were asked to avoid coffee, alcohol, and non-steroidal anti-inflammatory drugs at least 24 h prior to any trial. Baseline testing consisted of a muscle function assessment and a maximal aerobic capacity (VO_2_max) test.

After 56 days of supplementation, the participants reported to the lab to repeat all the muscle function assessments. The following day, participants performed a downhill run to induce muscle damage. The downhill run was performed on a modified TMX 3030c (Trackmaster, Newton, KS, USA) at a –15% grade. Prior to the downhill run, participants warmed up for five minutes at a 0% grade at a speed equivalent to 65% VO_2_Max. American College of Sports Medicine metabolic calculations for the estimation of energy expenditure during running [21] were utilized to determine the treadmill speed—on a level grade—that would approximately elicit 65% VO_2_max. Gas exchange was continuously measured (Parvo Medics, Sandy, UT, USA), and the treadmill speed was adjusted after two minutes to match 65% VO_2_Max. After the five-minute warm-up, participants completed a 45-min downhill run at a –15% grade and speed equivalent to 65% VO_2_Max. Muscle function was assessed one hour after completion of the downhill running bout. Participants returned to the lab at 24 h, 48 h, and 72 h following the downhill run for follow-up muscle function testing.

### 2.3. Supplementation

The investigational product (CurcuWIN^®^; OmniActive Health Technologies Ltd., Mumbai, India) contained turmeric extract (20–28%), a hydrophilic carrier (63–75%), cellulosic derivatives (10–40%), and natural antioxidants (1–3%) [19]. During the consent and familiarization process, participants were educated on which foods contained turmeric, and were asked to avoid those foods for the duration of the study while maintaining their normal diet. Participants were randomly assigned in a double-blind manner to either a placebo (corn starch, PLA), low dose of curcumin (50 mg of curcuminoids = 250 mg CurcuWIN^®^), or high dose of curcumin (200 mg of curcuminoids = 1000 mg CurcuWIN^®^) supplementation. Commercially available natural curcumin contains three curcuminoids in the following ratios: curcumin (71.5%), demethoxycurcumin (19.4%), and bisdemethoxycurcumin (9.1%) [22]. The day following baseline testing, participants were asked to ingest one dose with breakfast, lunch, and dinner for a total of three doses per day. To assist in blinding, all the doses required the ingestion of one capsule that was identical in shape, size, and color. As part of compliance monitoring to the supplementation regimen, participants were provided the capsules on a weekly basis in a pill container that provided seven doses and were asked to return the empty containers. A side effects questionnaire was also completed when receiving capsules. Compliance was set at ≥80%, and participants not meeting compliance were removed from the study.

### 2.4. Muscle Function Assessment

Muscle function was determined by assessing isokinetic and isometric peak torque as well as isokinetic power using a Biodex dynamometer (System 3; Biodex Medical Systems, Shirley, NY, USA) on the participant’s self-reported dominant leg. All the participants were familiarized with the testing protocols during his or her familiarization visit prior to supplementation. Before muscle function assessment, participants performed a five-minute self-paced warm up on a treadmill. Then, each participant was seated with their knee aligned with the lever arm axis of the dynamometer. The dynamometer warm-up consisted of three concentric-only extension and flexion repetitions at 50% of perceived maximal force production. Following the warm-up, participants were given a 90-s recovery.

Isokinetic peak torque and power were tested at a speed of 60° * s^−1^ through a modified range of motion, which began with their leg at approximately 120° knee flexion and continued through full extension of the knee (180° knee flexion). Once in the starting position, participants began each set of repetitions by forcefully extending their leg against the resistance through the full range of motion before forcefully flexing their knee back to the starting position. Participants repeated this motion for five continuous repetitions at maximal effort. Peak torque and power values for both extension and flexion were recorded as individual peak values and as average values across all the completed repetitions. After the isokinetic exercise, participants were given a three-minute rest. Then, isometric strength was assessed by three maximal voluntary extensions of the knee, each lasting five seconds against a fixed resistance arm at an angle of 120°. A one-minute rest was given between each repetition, and all the repetitions were performed at maximal effort. The highest isometric torque values were recorded as peak isometric torque in foot–pounds (ft–lbs) of torque, and the average peak torque value was also computed. Acute assessments of both isokinetic and isometric force and power are regularly used to assess acute changes in both dynamic and static force production in response to various forms of exercise stimuli [23,24]. Test–retest reliability using similar isokinetic devices with similar protocols and controls consistently yielded high measures of reliability and coefficients of variation less than 5% [25].

As an indirect indicator of muscle damage, perceived levels of anterior, posterior, and total soreness of the knee extensors were assessed by all participants using a 100-cm visual analog scale. Soreness was assessed along a 100-mm scale (0 mm = no soreness, 100 mm = extreme soreness) for each time point (pre-exercise, immediately (0 h), 24 h, 48 h, and 72 h post-exercise by drawing a line perpendicular to the continuum line extending from 0 mm to 100 mm. Soreness was evaluated by measuring the distance of each mark from 0 and rounded up to the nearest millimeter.

### 2.5. Statistical Analysis

Statistical analyses were performed using SPSS V.25 (IBM Corporation; Armonk, NY, USA). Primary outcomes were identified as peak torque values, while secondary outcomes were identified as average torque and power assessments. All the outcome measures were initially analyzed by a repeated measures analysis of variance (ANOVA) with three factors: gender (two levels), treatment (three levels), and time (six levels). Normality was assessed using the Shapiro–Wilk test with several variables violating the normality assumptions. All the non-normal variables were transformed using log_10_ before completing ANOVA procedures. All the data reported throughout is the raw data. Homogeneity of variance was assessed using Mauchly’s test of sphericity. When the homogeneity of variance assumption was violated, the Greenhouse–Geisser correction was applied when epsilon was <0.75, and the Huynh–Feldt correction was applied when epsilon was >0.75. Outside of expected outcomes (greater strength), gender contributed little to the overall model and did not impact treatment analysis; thus, gender was removed from the model and 3 (group) × 6 (time) mixed factorial ANOVA with repeated measures on time were subsequently used to assess the differences across time between groups. Tukey post hoc procedures were used when a significant finding (*p* ≤ 0.05) or trend (0.05 < *p* ≤ 0.10) was identified. Significant main effects for time were fully decomposed by completing factorial ANOVA with repeated measures on time. If significant differences in time or trends were identified, pairwise comparisons with Bonferroni corrections applied to the confidence interval were evaluated to determine between time points for each condition.

## 3. Results

### 3.1. Isokinetic Peak Flexion Torque

**Figure 2:**
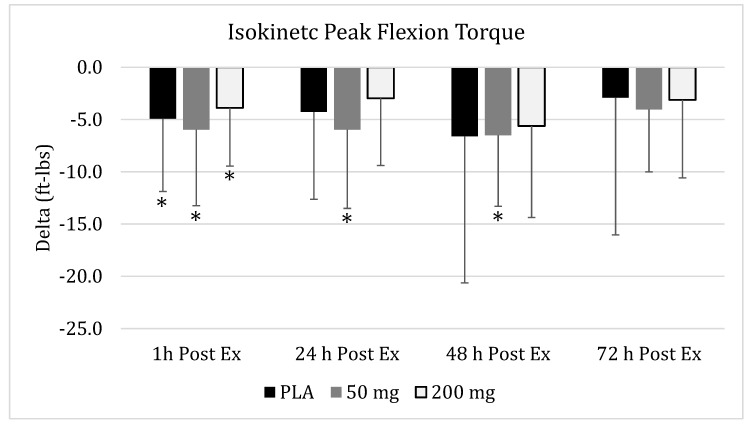
**Isokinetic Peak Flexion Torque.** Changes in isokinetic peak flexion torque as measured via an isokinetic dynamometer at 1 h, 24 h, 48 h, and 72 h post-muscle damaging exercise, downhill run, in placebo (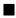), 50 of mg CUR (in the form of 250 mg of CurcuWIN^®^) (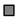), and 200 mg of CUR (in the form of 1000 mg of CurcuWIN^®^) (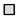). * = Significantly different than respective baseline value.

Using mixed factorial ANOVA, no significant group x time interaction (*p* = 0.60) effects were realized, while a significant main effect for time (*p* < 0.001) was identified for changes in peak flexion torque values (Figure 2). Regarding PLA, no significant changes across time (*p* = 0.15) were identified, while significant changes across time were found for the 50-mg dose (*p* = 0.03), and the 200-mg dose tended (*p* = 0.052) to exhibit changes across time. In the 50-mg dose group, peak flexion torque was significantly reduced in comparison to baseline values after one hour (95% CI: 2.46–9.48, *p* = 0.02), 24 h (95% CI: 2.34–9.60, *p* = 0.03), and 48 h (95% CI: 3.25–9.79, *p* = 0.02). In the 200-mg dose group, peak flexion torque was significantly reduced against baseline values after one hour (95% CI: 1.42–6.36, *p* = 0.04), and returned to baseline values for all the other time points.

### 3.2. Isokinetic Average Peak Flexion Torque

No significant group × time interaction (*p* = 0.55) effects were realized, while a significant main effect for time (*p* < 0.001) was identified for changes in the average peak flexion torque. A statistical tendency for change was observed in both the PLA (*p* = 0.08) and 200-mg (*p* = 0.07) groups, while values in the 50-mg dose (*p* = 0.03) exhibited a significant change across time. Pairwise comparisons in both the PLA (95% CI: 2.47–9.04, *p* = 0.046) and 200-mg (95% CI: 2.16–6.86, *p* = 0.006) groups indicated that the average peak flexion torque values were reduced one hour after damage in comparison to baseline values, but returned to baseline values after that initial drop in performance. Values in the 50-mg group exhibited more changes, with significantly reduced values being observed one hour (95% CI: 2.23–9.40, *p* = 0.03) and 48 h (95% CI: 2.45–8.57, *p* = 0.04) after damage, with values 24 h after damage tending to be reduced (95% CI: 1.58–9.48, *p* = 0.06).

### 3.3. Isokinetic Peak Extension Torque

**Figure 3:**
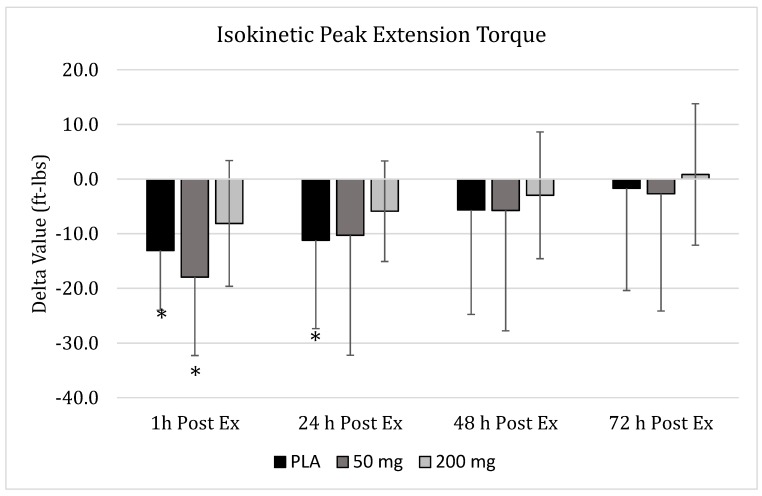
**Isokinetic Peak Extension Torque.** Changes in isokinetic peak extension torque as measured via an isokinetic dynamometer 1 h, 24 h, 48 h, and 72 h post-muscle damaging exercise, downhill run, in placebo (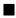), 50 mg of CUR (in the form of 250 mg of CurcuWIN^®^) (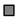), and 200 mg of CUR (in the form of 1000 mg of CurcuWIN^®^) (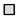). * = Significantly different than respective baseline value.

Using mixed factorial ANOVA, no significant group x time interaction (*p* = 0.52) effects were realized, while a significant main effect for time (*p* < 0.001) was identified for changes in peak extension torque values (Figure 3). Values changed significantly across time for the PLA (*p* = 0.002) and 50-mg (*p* = 0.04) groups, while no significant change was exhibited for the 200-mg dose (*p* = 0.16). In PLA, peak extension torque was significantly reduced one hour (95% CI: 8.10–18.04, *p* = 0.01) and 24 h (95% CI: 3.84–18.56, *p* = 0.03) after completion of the downhill running bout in comparison to pre-damage values. Changes in the 50-mg group indicated that peak extension torque in comparison to baseline values were significantly reduced (95% CI: 11.02–24.85, *p* < 0.001) one hour after completion of the damaging exercise, but returned to baseline values for all the other time points. No significant changes across time (*p* = 0.159) were observed for the 200-mg dose.

### 3.4. Isokinetic Average Peak Extension Torque

Changes in average peak extension torque values indicated no significant group x time interaction (*p* = 0.578) in conjunction with a significant main effect for time (*p* < 0.001). Individual pairwise comparisons within each condition between individual time points revealed a similar pattern of change. In this respect, significant changes across time were found for PLA (*p* < 0.001), 50 mg (*p* = 0.03), and 200 mg (*p* = 0.03). Pairwise comparisons for PLA indicated that average peak extension torque values were significantly lower one hour (95% CI: 6.83–18.88, *p* = 0.02) after the running bout when compared to baseline levels. Individual comparison in the 50-mg group revealed that values were significantly lower than baseline after one hour (95% CI: 9.72–24.39, *p* <0.001) and 24 h (95% CI: 3.48–23.07, *p* = 0.04). Similarly, changes within the 200-mg group exhibited a significant reduction from baseline after one hour (95% CI: 6.59–16.37, *p* = 0.002).

### 3.5. Isokinetic Peak Extension Power

**Figure 4:**
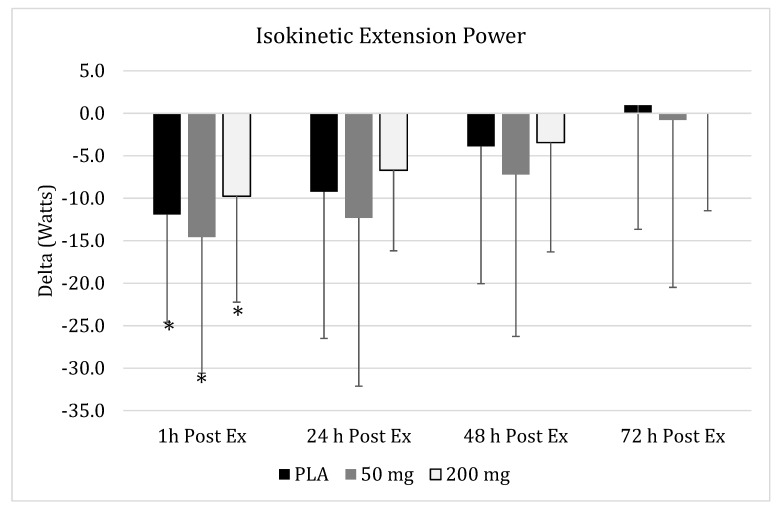
**Isokinetic Extension Power.** Changes in isokinetic extension power as measured via an isokinetic dynamometer 1 h, 24 h, 48 h, and 72 h post-muscle damaging exercise, downhill run, in placebo (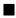), 50 mg of CUR (in the form of 250 mg of CurcuWIN^®^) (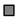), and 200 mg of CUR (in the form of 1000 mg of CurcuWIN^®^) (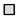). * = Significantly different than respective baseline value.

No significant group x time interaction (*p* = 0.39) effect was found for changes in peak extension power in all the supplementation groups against time (Figure 4). A significant main effect over time was found (*p* = 0.002). Within-group changes in the PLA group revealed a tendency for values to change (*p* = 0.08). Individual pairwise comparisons in PLA indicated that peak extension power data was significantly reduced (95% CI: 6.16–17.69, *p* = 0.04) one hour after downhill treadmill running. Similarly, within-group changes in the 50-mg group indicated a statistical trend (*p* = 0.051) for peak extension power values to change across time. Again, pairwise comparisons revealed that the only significant changes for peak extension power occurred one hour (95% CI: 6.86–22.30, *p* = 0.03) after completion of the treadmill exercise bout. Changes in the 200-mg group also exhibited a tendency for power values to change across time (*p* = 0.09), with individual pairwise comparisons indicating that values one hour (95% CI: 4.24–15.29, *p* = 0.03) after completion of the damage bout were significantly lower than baseline, while changes after 24 h (95% CI: 2.51–10.91, *p* = 0.09) tended to be lower.

### 3.6. Isokinetic Peak Flexion Power

**Figure 5:**
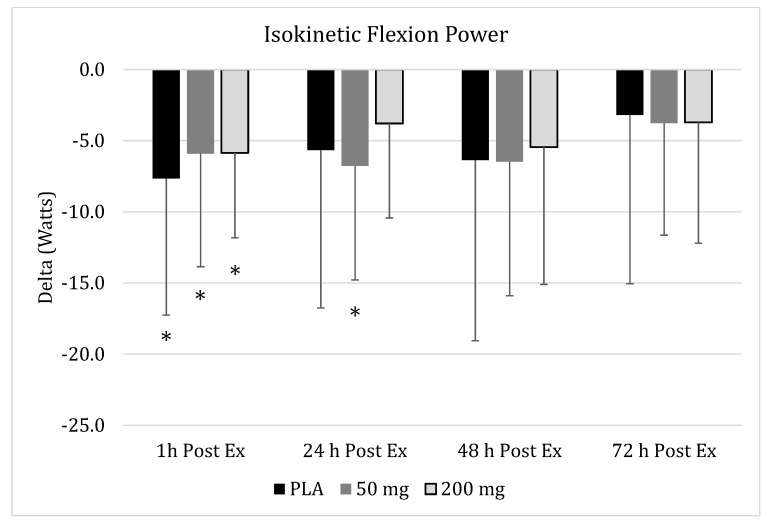
**Isokinetic Flexion Power.** Changes in isokinetic peak flexion power as measured via an isokinetic dynamometer 1 h, 24 h, 48 h, and 72 h post-muscle damaging exercise, downhill run, in placebo (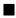), 50 mg of CUR (in the form of 250 mg of CurcuWIN^®^) (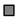), and 200 mg of CUR (in the form of 1000 mg of CurcuWIN^®^) (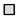). * = Significantly different than respective baseline value.

No significant group x time interaction (*p* = 0.96) effect was found in any of the supplementation groups for peak flexion power (Figure 5). A significant main effect over time was found (*p* < 0.001). Within-group changes in the PLA group revealed no significant change across time (*p* = 0.14), while values in 50-mg group revealed a significant within-group change (*p* = 0.049), while values for the 200 mg tended to change (*p* = 0.08). Significant reductions from baseline in peak flexion power were observed one hour after running for both PLA (95% CI: 3.29–12.03, *p* = 0.03) and 200 mg (95% CI: 3.21–8.51, *p* = 0.01), while values in the 50-mg group were significantly reduced from baseline after one hour (0.001–0.088, *p* = 0.04) and 24 h (95% CI: 0.85–6.74, *p* = 0.03), and tended to be lower after 48 h (95% CI: 1.18–9.73, *p* = 0.08).

### 3.7. Isometric Peak Torque

No significant group x time interaction (*p* = 0.57) was found, while a significant main effect over time was found (*p* = 0.046). Within-group changes in the PLA group revealed no significant change across time (*p* = 0.43) while values in both the 50-mg (*p* = 0.01) and 200-mg groups (*p* = 0.02) significantly decreased across time. As expected, no individual pairwise comparisons within PLA revealed any statistically significant changes (*p* > 0.05). Significant reductions from baseline in isometric peak torque were observed one hour (95% CI: 8.73–19.16, *p* < 0.001) and 24 h (95% CI: 7.95–23.62, *p* = 0.004) in the 50-mg group. Within the 200-mg group, isometric peak torque values were significantly reduced 24 h after exercise (95% CI: 1.69–13.79, *p* = 0.02), but were similar to the baseline values at all the other time points. 

### 3.8. Isometric Average Peak Torque

No significant group x time interaction (*p* = 0.52) was found, while a significant main effect over time was found (*p* = 0.001) for isometric average peak torque values. Within-group changes in the PLA (*p* = 0.06) and 50-mg groups (*p* = 0.11) were not different from baseline, while changes in the 200-mg group exhibited significant reductions across time (*p* = 0.02) with no individual pairwise comparisons reaching statistical significance.

### 3.9. Perceived Soreness

**Figure 6:**
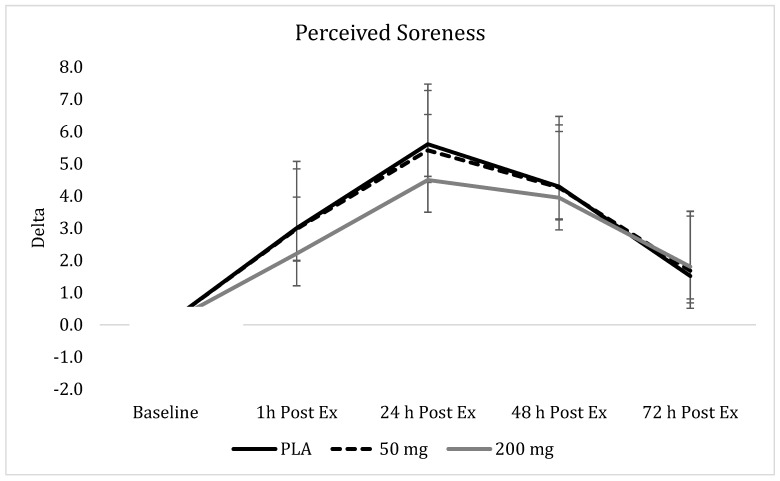
**Perceived Total Thigh Soreness.** Changes in perceived total thigh soreness 1 h, 24 h, 48 h, and 72 h post-muscle damaging exercise, downhill run, in placebo (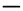), 50 mg of CUR (in the form of 250 mg of CurcuWIN^®^) (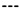), and 200 mg of CUR (in the form of 1000 mg of CurcuWIN^®^) (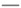).

Statistically significant main effects for time were identified, indicating that perceived thigh soreness levels significantly increased in response to the exercise bout. Anterior thigh soreness (*p* < 0.001), posterior thigh soreness (*p* < 0.001), and total thigh soreness (*p* < 0.001) were all found to significantly increase across time. No significant group x time interaction were identified for anterior thigh soreness (*p* = 0.73), posterior thigh soreness (*p* = 0.73), and total thigh soreness (*p* = 0.30). Non-significant improvements in exercise-induced total thigh soreness indicated that the 200-mg groups reported 26%, 20%, and 8% less soreness immediately, 24 h, and 48 h after exercise, respectively, than the soreness levels that were reported in the PLA and 50-mg groups. However, these differences failed to reach statistical significance (Figure 6).

## 4. Discussion

The main findings of this study indicate widespread decreases in torque and power after completion of a one hour bout of downhill running. Responses in both the PLA and 200-mg groups were largely similar across time, while the 50-mg dose failed to respond in a similar fashion for any measured variable in comparison to either PLA or the 200-mg dose. In particular, isokinetic peak extension torque values experienced significant reductions in PLA and 50 mg across time, while no changes were found in the 200-mg group. Several other measured variables indicated that the 200-mg and PLA groups responded in a similar fashion with the smallest decrements in performance occurring in the 200-mg group, while changes in both of these groups were more favorable than those outlined in the 50-mg group. Additionally, a single bout of downhill treadmill running led to a widespread significant increases in perceived muscle soreness, but no differences between groups were identified.

Despite the proven preclinical efficacy, curcumin and other curcuminoids are known for poor solubility, low absorption from the gut, rapid metabolism, and rapid systemic elimination, which contribute to an overall low oral bioavailability [26]. The curcumin used in the current study has been shown to have a 45.9-fold higher absorption over standard curcumin [19]. Notably, the form of curcumin used in the current study has previously been shown to illustrate a clinically meaningful improvement in endothelial function, as measured by flow-mediated dilation when a dose of 200 mg of curcuminoids was delivered [20]. Curcumin has previously been shown to increase vasodilation similar to exercise [27], and curcumin ingestion with aerobic exercise training is more effective than curcumin ingestion or aerobic exercise training alone at reducing left ventricular afterload [28]. In addition, curcumin inhibits the conversion of amino acids from muscle into glucose (gluconeogenesis) [14]. From a performance and muscle damage recovery perspective, limited work is available regarding the role of curcumin. In an animal running model, curcumin (10-mg pellet added to food for three consecutive days) was shown to significantly improve running time to exhaustion and voluntary physical activity in rats exposed to a single bout of downhill running [9]. Additionally, curcumin treatment in the downhill running rats also improved circulating levels of tumor necrosis factor-alpha to similar levels as those of the placebo within 48 h of completing the treadmill running [9]. In addition, Vitadell et al. [29] injected rats with curcumin and treated them to either hindlimb unloading or standard caging. After seven days, curcumin treatment significantly attenuated the loss of soleus mass and the myofiber cross-sectional area, while also sustaining a greater maintenance of force production attributes. Additionally, curcumin treatment was found to significantly blunt changes in protein and lipid oxidation in unloaded, treated rats compared to untreated ones. In one of the few studies conducted in humans to examine the impact of curcumin treatment on recovery from damaging exercise, Nicol et al. [12] had 17 men supplement in a double-blind, placebo controlled fashion with either curcumin (5000 mg of curcuminoids per dose) for 2.5 days leading up to and 2.5 days after completing an eccentric exercise protocol. Jump performance, creatine kinase, and pain indicators were assessed along with changes in circulating cytokines. It was concluded that curcumin reduced pain associated with soreness and may improve jumping performance in the days following muscle damage. These outcomes are in accordance with the changes seen in peak extension torque values in the present study, but are not entirely supported with the reported soreness levels in the present study. While the most favorable pattern of change was observed in the 200-mg group (Figure 6), these changes failed to reach a statistically significant levels in comparison to PLA and the 50-mg dose. Briefly, the 200-mg curcumin dose exhibited no loss of torque production, while significant reductions were noted one hour and 24 h after completion of the damage bout in the PLA group (Figure 3). Changes in peak flexion torque values revealed a similar pattern of change for the PLA and 200-mg group, with immediate significant reductions in peak flexion torque, but a return to baseline values by the next time point at 24 h. Unexpectedly, peak flexion torque values were still significantly reduced at 24 h and 48 h (Figure 2) after completion of the damage bout in the 50-mg group. These divergent changes seemingly suggest that a dose–response action may be evident between the ability of curcumin to mitigate losses of force production after damaging exercise. A similar pattern of change was observed for total thigh soreness, with the 200-mg dose exhibiting smaller perturbations in reported soreness, but the magnitude of these values failed to reach statistical significance. Similar patterns of change were noted for all the other variables, with significant reductions occurring in all the groups for isokinetic extension and flexion power (Figure 4 and Figure 5, respectively).

Several strengths and limitations are present in this study that require discussion. For starters, the current study was the first investigation in humans to examine the ability of different dosages of curcumin (50 and 200 mg of curcuminoids) to mitigate recovery from damaging exercise using a novel formulation that has been previously shown to optimize bioavailability [19]. In addition, at eight weeks, the current investigation was one of the longer curcumin supplementation studies to date with the majority of studies supplementing in varying amounts for 1 to 4 weeks. A key limitation of the supplementation protocol used in the present study was the ceasing of supplementation after damage. While speculative, it remains possible that continuing supplementation through all the recovery time points may have provided better support to mitigate the changes observed, as this has been observed and highlighted to be a confounding factor surrounding research of this nature [5,25]. This consideration may also help to explain why various mean changes were observed in multiple outcome measures, but the overall magnitude of change failed to yield statistical significance. Beyond these points, the already limited research in humans becomes even more convoluted when study cohorts comprising differing demographics and different forms of exercise are utilized. In particular, the present study is the only investigation to introduce data featuring females. While the influence of gender in the present study was considered to be statistically irrelevant, known differences exist between how males and females respond to damaging exercise [30]. As such, these confounding influences could seemingly interact with the known ability of curcumin to change the observed responses to inflammation and oxidative stress. Finally, it is challenging to understand why changes in peak extension torque reached statistically significant thresholds in the 200-mg dose, but such changes failed to be realized when investigating the changes seen in peak flexion torque from the same exercise bout. While merely speculative, these changes may be due to a greater magnitude of stress placed on the knee extensors throughout the downhill running bout as opposed to the knee flexors, and this greater stress was differentially impacted by the curcumin supplementation. Irrespective of these differences, results from the previous study demonstrate consistently better performance outcomes in the higher dose of curcumin (200 mg) when compared to the lower dose (50 mg). In a somewhat unexpected fashion, the placebo group responded more favorably than the 50-mg dosage and in all but one variable (isokinetic peak extension torque), while the PLA group responded similarly to the 200-mg group. It is possible that a certain threshold of curcumin (or curcuminoids) is needed to exert any impact on our measured outcomes, and consequently, the 200-mg dose crossed this threshold, while the 50-mg dose did not. When considered in combination with the cessation of supplementation after conclusion of the damage bout, the lack of circulating curcuminoids in the 50-mg dose (versus the 200-mg dose) is considered to be the most likely reason for our dichotomous outcomes from a dose perspective. Another key consideration when interpreting our results in comparison to previous findings are the different patterns of exercise stress and damage that are employed. In the present study, a downhill running protocol was utilized, which is an established protocol to instigate metabolic as well as mechanical stress [6]. Other damage models such as the one utilized in the Nicol protocol [12], employ high volumes of resistance exercise contractions that involve eccentric contractions resulting in high levels of mechanical overload on the involved musculature, and have been clearly established as causing muscle damage [6].

## 5. Conclusions

In conclusion, results from the present study highlight the ability of a high dose of CurcuWIN^®^ (1000-mg dose delivering 200 mg of curcuminoids) to prevent the observed decreases in peak extension torque values seen one and 24 h after muscle-damaging exercise. In comparison, a lower dose of CurcuWIN^®^ (delivering 50 mg of curcuminoids) was unable to attenuate performance changes to a similar pattern as what was observed in PLA. While this study investigated changes in performance, future studies should also investigate more objective measures (blood markers such as creatine kinase or myoglobin) of muscle damage in cohorts of trained and untrained samples. 

## Figures and Tables

**Table 1:** Subject characteristics at baseline. Data is presented as means ± standard deviation. No significant differences between groups were found at baseline (*p* > 0.05).

	Placebo	50 mg	200 mg
**Age [years]**			
Women (*n* = 11/10/11)	20.8 ± 1.5	21.5 ± 3.0	21.5 ± 2.7
Men (*n* = 10/10/11)	20.8 ± 2.1	21.3 ± 1.8	21.6 ± 1.9
Total (*n* = 21/20/22)	20.8 ± 1.8	21.4 ± 2.4	21.6 ± 2.3
**Height [cm]**			
Women (*n* = 11/10/11)	165.1 ± 5.3	167.6 ± 5.5	162.6 ± 7.8
Men (*n* = 10/10/11)	176.6 ± 6.4	178.8 ± 8.8	175.9 ± 5.0
Total (*n* = 21/20/22)	170.6 ± 8.2	173.2 ± 9.2	169.3 ± 9.4
**Weight [kg]**			
Women (*n* = 11/10/11)	62.2 ± 6.0	61.6 ± 10.2	58.2 ± 8.6
Men (*n* = 10/10/11)	78.2 ± 9.9	82.8 ± 11.5	78.2 ± 11.6
Total (*n* = 21/20/22)	69.8 ± 11.3	72.2 ± 15.2	68.2 ± 14.3
**Body Mass Index (kg/m^2^)**			
Women (*n* = 11/10/11)	22.8 ± 1.8	21.9 ± 3.2	21.9 ± 1.9
Men (*n* = 10/10/11)	25.1 ± 3.0	25.9 ± 2.6	25.2 ± 3.3
Total (*n* = 21/20/22)	23.9 ± 2.6	23.9 ± 3.5	23.6 ± 3.1
**Maximum Aerobic Capacity [VO_2_mMax in mL/kg/min]**			
Women (*n* = 11/10/11)	39.2 ± 4.9	39.4 ± 4.7	40.9 ± 7.7
Men (*n* = 10/10/11)	44.4 ± 6.0	47.4 ± 7.4	46.3 ± 6.0
Total (*n* = 21/20/22)	41.6 ± 6.0	43.2 ± 7.2	43.6 ± 7.3
**Race**			
White	16 (10F, 6M)	15 (7F, 8M)	19 (10F, 9M)
Asian	3 (2 F, 1M)	2 (1F, 1M)	1 (1M)
African-American	1 (1M)	1 (1M)	-
Latino	1 (1M)	2 (2F)	2 (1F, 1M)
